# Medication-overuse headache: causes, consequences and management

**DOI:** 10.1007/s00415-021-10720-5

**Published:** 2021-08-05

**Authors:** Thomas H. Massey, Neil P. Robertson

**Affiliations:** Department of Neurology, Division of Psychological Medicine and Clinical Neuroscience, Cardiff University, University Hospital of Wales, Heath Park, Cardiff, CF14 4XN UK

## Introduction

Medication-overuse headache (MOH) has a prevalence of approximately 1% and is among the top 20 causes of disability worldwide, leading to considerable medical, social, and economic costs. It is defined in the International Classification of Headache Disorders (ICHD-3) as a headache occurring on at least 15 days per month in someone with a pre-existing headache disorder and developing as a result of regular overuse of one or more acute/symptomatic headache treatments for at least 3 months. Overuse is defined as at least 10 days per month for triptans, opioids and ergotamine, or at least 15 days per month for paracetamol, aspirin, and non-steroidal anti-inflammatory drugs. The frequency of acute medication use seems more important in the development of MOH than the cumulative dosage taken. MOH most often develops in those with underlying migraine or tension-type headache, but it can occur with other primary headache disorders such as cluster headache or new daily persistent headache. The quality of the headache is often similar to that of the underlying headache disorder which can make diagnosis of MOH difficult unless symptomatic improvement after acute drug withdrawal is observed. Women are four times as likely to develop MOH as men with peak prevalence at age 40–50. Other risk factors associated with MOH include obesity, anxiety or depression, low educational level, smoking, chronic musculoskeletal or gastrointestinal diseases, and physical inactivity. Treatment strategies include drug withdrawal, with or without preventive medication cover, and patient education.

This month’s journal club examines three papers reporting on the causes, consequences and treatments of MOH. The first is a systematic review and critical appraisal of gene polymorphism association studies, assessing the evidence for genetic predisposition to MOH. The second examines association between MOH and suicidal ideation and the third reports an open-label, randomized clinical trial of three treatment strategies.

## A systematic review and critical appraisal of gene polymorphism association studies in medication-overuse headache

The risk of developing MOH is increased in those with a family history of MOH or substance abuse. Since the risk of addictive behaviours in individuals is at least partly genetic, multiple studies have looked for associations between genetic polymorphisms and risk of MOH. This systematic review collated and analysed 17 such studies with sample sizes between 22 and 227.

Taking a candidate variant approach, two associations with susceptibility to MOH survived correction for multiple testing: a single nucleotide polymorphism in the *RAMP1* (receptor activity modifying protein 1) gene and a 48 bp variable number tandem repeat in the *DRD4* (dopamine receptor D4) gene. A small number of variants in genes involved in drug dependence pathways or the dopaminergic system were also associated with clinical features or outcomes of MOH. For example, polymorphisms in *WFS1* (wolfram syndrome 1) or *BDNF* (bone-derived natriuretic factor) were associated with significantly greater acute drug consumption, and a polymorphism in COMT (catechol-O-methyltransferase) was associated with reduced risk of relapse into MOH 1 year after successful acute drug withdrawal.

**Comment:** Although there are significant associations between MOH features and some genetic polymorphisms, they have all come from candidate gene analyses in very small study populations. Candidate genes have been chosen on the basis of presumed likely pathogenic pathways in MOH relating to addictive behaviours rather than on any prior evidence. Given the prevalence of MOH, an unbiased genome-wide association study in a large population should be possible and informative. Variables in phenotype such as the underlying primary headache disorder, the medication overused, and drug frequency and dosage will need to be carefully controlled.

*Cargnin, S. et al. (2018) Cephalalgia 38: 1361–1373*

## Association between suicidal risks and medication-overuse headache in chronic migraine: a cross-sectional study

MOH involves elements of both chronic pain and substance misuse, both of which have been linked to suicide risk. This study aimed to test the association between MOH and suicidal ideation and/or attempt. A total of 603 consecutive patients newly diagnosed with chronic migraine in a specialist headache clinic (Taipei Veterans Hospital) were recruited, 320 with concomitant MOH and 283 without. Patients were aged between 20 and 65 (mean age 42.0 years) with a female:male ratio of 4:1, reflecting the gender distribution of chronic migraine.

There were significant associations between the presence of MOH and lower education levels and earlier onset of migraine, and more frequent analgesic use. However, MOH was not associated with a higher number of headache days per month nor the severity of depression, anxiety or poor sleep. In the study population, 214 (35.5%) had experienced suicidal ideation and 81 (13.4%) had made a suicide attempt at some point in their lives. Patients with chronic migraine and MOH were significantly more likely to have had suicidal ideation (41.6% vs 29.4%; *p* = 0.002) or made a previous suicide attempt (16.7% vs 10.1%; *p* = 0.018) than those with chronic migraine alone. The associations remained significant after controlling for demographics, headache profile, depression, anxiety, and sleep quality. Overall, the presence of MOH in chronic migraineurs was associated with increased risks of suicidal ideation (odds ratio 1.75, 95% CI 1.20–2.56) and suicide attempt (odds ratio 1.88, 95% CI 1.09–3.24).

**Comment:** In patients with chronic migraine, MOH is associated with greater suicidal risks. This study was well powered, took consecutive patients from a specialist centre to reduce selection bias, and controlled for a number of putative confounders. It was not designed to address whether MOH causes the increased suicide risks observed. The study did not take into account treatments being used for chronic migraine, the type or dosage of medication involved in the MOH, nor the presence or absence of substance misuse disorders, each of which is a potential source of variability or bias in the data.

*Wang, Y.-F. et al. (2021) J. Headache and Pain, 22: 36.*

## Comparison of 3 treatment strategies for medication-overuse headache. A randomized clinical trial

This paper reported an open-label, randomised clinical trial of three MOH outpatient treatment strategies. A total of 120 consecutive patients with MOH were recruited from a tertiary headache centre in Denmark, randomised 1:1:1 to three treatment strategies, and followed up for 6 months. Inclusion criteria included a headache diagnosis of chronic migraine (52.0%), tension-type headache (15.7%) or a mixture of the two (32.4%). Individuals with other headache diagnoses (e.g. cluster headache) or significant physical or psychiatric comorbidities were excluded from the study.

Treatment strategies were (1) withdrawal alone; (2) withdrawal with preventive treatment from start; (3) preventive treatment without withdrawal. Drug withdrawal involved discontinuation of all analgesics for 2 months. The withdrawal group (1) was offered preventive medication after the initial 2-month withdrawal period. Preventive medications were prescribed using existing Danish guidelines and included candesartan (44.1%), amitriptyline (12.7%), and metoprolol (9.8%). The mean age of patient was 43.9 years, and the female: male ratio was 4:1, as expected. The medications overused were simple analgesics (28.4%), triptans (17.6%), or combinations of these and other acute drugs (53.9%), with the duration of overuse ranging from 3 months to over 10 years. On entering the study, patients had a median of 27 headache days per month (range 15–30 days). During the withdrawal period, short-term analgesics were completely discontinued by 20 of 36 (55.6%) in the withdrawal group (1) and 18 of 31 (58.1%) in the withdrawal plus preventive group (2), with the remaining patients reducing their intake of short-term medications to below the threshold for MOH.

After 6 months, there was a reduction in headache days per month in each of the three treatment groups [withdrawal − 8.5 days (95% CI 5.6–11.5); withdrawal plus preventive − 12.3 days (95% CI 9.3–15.3); preventive alone − 9.9 days (95% CI 7.2–12.6)] with no significant difference between the three groups. An analysis of secondary endpoints at 6 months showed that the withdrawal plus preventive strategy conferred a modest but significant advantage over the other two strategies. For example, withdrawal plus preventive was 80% more likely to promote conversion from chronic to episodic headache than withdrawal alone (relative risk 1.8 (95% CI, 1.1–2.8; *p* = 0.03).

**Comment:** This pragmatic study directly compared three simple outpatient strategies for treating MOH and showed that they are all effective, with withdrawal plus preventive being marginally favoured. Limitations of this study included the small sample sizes, the lack of a no treatment group, and the heterogeneity of the population. For example, the underlying headache diagnosis, the medications overused, the duration of MOH, and the preventives prescribed were all variable. The small sample sizes prevented subgroup analyses to assess the impact of these factors.

*Carlsen, L.N. et al. (2020) JAMA Neurol. 77: 1069–1078*

## Conclusion

MOH is common and debilitating, has a widely variable phenotype, usually arises as a result of inadequate long-term treatment of an underlying headache disorder and can be associated with an increased suicide risk. The aetiology of this condition remains unclear, but any candidate gene associations are likely to require confirmation in an unbiased genome-wide association study. Drug withdrawal strategies are effective but need to be coupled with patient education on the risks of analgesic overuse to prevent relapse.

